# NQO-Induced DNA-Less Cell Formation Is Associated with Chromatin Protein Degradation and Dependent on A_0_A_1_-ATPase in *Sulfolobus*

**DOI:** 10.3389/fmicb.2017.01480

**Published:** 2017-08-14

**Authors:** Wenyuan Han, Yanqun Xu, Xu Feng, Yun X. Liang, Li Huang, Yulong Shen, Qunxin She

**Affiliations:** ^1^Archaea Centre, Department of Biology, University of Copenhagen Copenhagen, Denmark; ^2^State Key Laboratory of Agricultural Microbiology and College of Life Science and Technology, Huazhong Agricultural University Wuhan, China; ^3^State Key Laboratory of Microbial Resources, Institute of Microbiology, Chinese Academy of Sciences Beijing, China; ^4^State Key Laboratory of Microbial Technology, Shandong University Jinan, China

**Keywords:** *Sulfolobus*, DNA damage, DNA-less cell formation, chromatin protein, A_0_A_1_-ATPase

## Abstract

To investigate DNA damage response in the model crenarchaeon *Sulfolobus islandicus*, four different DNA damage agents were tested for their effects on cell death of this archaeon, including UV irradiation, methyl methanesulfonate, cisplatin, and 4-nitroquinoline 1-oxide (NQO). Cell death featured with DNA-less cell formation was revealed in DNA damage treatment with each agent. Cellular responses upon NQO treatment were characterized in details, and following sequential events were revealed, including: a modest accumulation of G1/S phase cells, membrane depolarization, proteolytic degradation of chromatin proteins, and chromosomal DNA degradation. Further insights into the process were gained from studying drugs that affect the archaeal ATP synthase, including a proton gradient uncoupler and an ATP synthase inhibitor. Whereas the proton uncoupler-mediated excess proton influx yielded cell death as observed for the NQO treatment, inhibition of ATP synthase attenuated NQO-induced membrane depolarization and DNA-less cell formation. In conclusion, the NQO-induced cell death in *S. islandicus* is characterized by proteolytic degradation of chromatin protein, and chromosomal DNA degradation, which probably represents a common feature for the cell death induced by different DNA damage agents.

## Introduction

All living organisms encounter various endogenous factors and environmental stimuli that damage their genetic material, yielding DNA lesions that should be repaired properly in order to maintain their genome integrity. Diverse DNA repair mechanisms have been evolved to deal with DNA damage, including base excision repair, nucleotide excision repair (NER), mismatch repair, and homologous recombination repair. Investigation of DNA lesion repair in bacteria and eukaryotes has revealed that these DNA repair activities are under the control of a signal transduction pathway named DNA damage response (DDR). Upon DNA damage, DDR regulates expression of genes that function in cell cycle arrest or diverse DNA repair pathways, and cell cycle only resumes after the completion of DNA damage repair (Giglia-Mari et al., 2011; Maréchal and Zou, 2013; Baharoglu and Mazel, 2014). Cell death is induced if the extent of DNA damage is beyond the repair capacity of the cell. For example, metazoans employ programmed cell death to eliminate cells containing a severely damaged genome in order to maintain the genome stability at the organismal level and avoid cancerogenesis (Elmore, 2007).

To date, there is only a rudimentary understanding of DNA damage repair in Archaea, the third domain of life. When the sequences of the first archaeal genomes became available about two decades ago, a number of genes coding for putative DNA repair proteins were identified. Many of them were expressed as recombinant proteins and characterized. These archaeal enzymes show the predicted activities (Grogan, 2004, 2015; Kelman and White, 2005; Grasso and Tell, 2014; Ishino and Narumi, 2015). Nevertheless, further investigations have revealed more complex picture of archaeal DNA repair mechanisms: (a) thermophilic archaea lack the classical mismatch repair enzymes that function in eukaryotes and most bacteria but code for a non-canonical mismatch repair pathway (Ishino et al., 2016; Castaneda-Garcia et al., 2017), (b) genetic studies of genes coding for putative NER enzymes show that they probably do not function in DNA repair in thermophilic archaea, including *Thermococcus kodakarensis*, a euryarchaeon (Fujikane et al., 2010) and *Sulfolobus islandicus*, a crenarchaeon (Zhang et al., 2013), and (c) the crenarchaeal genus *Sulfolobus* possesses a UV-inducible DNA transfer system implicated in DNA damage repair (Ajon et al., 2011; van Wolferen et al., 2015, 2016).

Here, we investigated DNA damage response in *S. islandicus*, an extremely thermophilic archaeon to four different DNA damage agents including UV irradiation, methyl methanesulfonate (MMS), cisplatin and 4-nitroquinoline 1-oxide (NQO) and found that, upon treatment at a lethal dosage, each drug induced DNA-less cell formation in this archaeon. Systematical analysis of NQO-induced cellular response revealed that the DNA-less cell formation is characterized with membrane depolarization, proteolytic degradation of chromatin proteins and chromosomal DNA degradation, a process that is probably triggered by the archaeal ATP synthase-mediated proton influx.

## Materials and Methods

### Growth Conditions and Drug Treatments

*Sulfolobus islandicus* Rey15A E233S1 (ΔpyrEFΔlacS) was grown in SCVU [0.2% sucrose, 0.2% Casamino acids, 5 μl/ml of vitamin mixture solution (Deng et al., 2009) and 20 μg/ml uracil] medium at 78°C. Balanced cultures (A_600_ = 0.2), which had been grown at exponential phase for at least 72 h, were used in all experiments. The drugs used in the experiments included NQO, MMS, cisplatin, carbonyl cyanide *m*-chlorophenylhydrazone (CCCP) (Sigma–Aldrich) and dicyclohexylcarbodiimide (DCCD) (Sigma–Aldrich). NQO was prepared in DMSO to a stock concentration of 130 mM and diluted to 1.3 mM with distilled water, while cisplatin was prepared to a stock concentration of 4 mg/ml. CCCP and DCCD were dissoved in DMSO to a stock concentration of 100 mM, and added in cultures at the final concentration of 40 μM. To start drug treatment, the drugs were added into cultures directly as indicated, and the cultures supplemented with corresponding amount of DMSO were set as control if applicable. UV treatment was performed with UV stratalinker 1800 (stratagene, United States). The treated cultures were grown at 78°C, protected from light, and cell samples were withdrawn at the indicated time points and subjected to further analysis.

### Cell Viability

Cells were collected by centrifugation and resuspended in 1 ml of fresh medium. The cell suspension was serially diluted and plated using a two-layer plating technique (Deng et al., 2009). Colonies appeared on plates after 6 days of incubation and counted, giving colony formation units (CFUs) per ml culture.

### Flow Cytometry

The cells samples for flow cytometry were prepared following the procedure described by Bernander and Poplawski (1997), with some modifications. 300 μl of *S. islandicus* culture was mixed with 700 μl of absolute ethanol and stored at 4°C for at least 12 h for cell fixation. Then, cells were collected by centrifuging at 2800 rpm for 20 min and resuspended in 1 ml of the wash buffer (10 mM Tris-NaCl, pH 7.5, 10 mM MgCl_2_), and collected again by centrifugation. Finally, the cell pellets were resuspended in 140 μl of staining solution [the washing buffer containing 40 μg/ml ethidium bromide (Sigma–Aldrich) and 100 μg/ml mithramycin A (Apollo Chemical)] and stained for at least 20 min on ice. Stained cells were analyzed in an Apogee A40 cytometer with a 405 nm laser, and a dataset of at least 60,000 cells was collected for each sample. For each cell, information of four parameters was collected, including FL1 (green fluoresence), FL2 (red fluoresence), FSC (forward scattered light), and SSC (side scattered light). When applicable, values of all the four parameters are shown in liner sacle. For the cells stained with ethidium bromide and mithramycin A, FL2 represents DNA content. In FL2 -SSC cytograms, the population of DNA-less is separated from those containing one or more chromosomes and thus can be quantified with Apogee Flow Hisogram.

### Membrane Permeability and Polarity Analyses

For membrane permeability analysis, cells were collected from each sample by centrifugation and washed with fresh medium of the same composition. Then, the cells were resuspened in 150 μl fresh medium containing 0.5 μl of dye mix of SYTO 9 and propidium iodide (PI) in the ratio 1:1 (from the LIVE/DEAD BacLight bacterial viability kit, Molecular Probes). After incubation for 15 min at room temperature in the dark, the cell samples were analyzed by flow cytometry. The intensity of green (FL1, SYTO9) and red (FL2, PI) fluoresence was measured with an Apogee A40 cytometer (Apogee Flow Systems) equipped with a 488 nm laser and the cell population that exhbited stonger red signal over green signal was quantified using the Apogee Flow Hisogram software as PI-postive cells.

For membrane polarity analysis, DiBAC4 (Sigma–Aldrich) was added to each cell suspension to the concentration of 0.5 μg/ml and incubated for 5 min in the dark. The flueroscence intensity (FL1) in individual cells was estimated in a similar way as for the membrane permeability analysis described above.

### DAPI Staining and Microscopy

Fixed cell samples prepared for flow cytometry were also used for DAPI analysis. Cell pellets were washed with 1 ml of the wash buffer and resuspended in 20 μl DAPI (Sigma) solution (the same buffer containing 3 μg/ml DAPI). After incubation on ice in the dark for at least 1 h, 1 μl of the cell suspension was transferred to a glass slide pre-coated with 30 μl of 1% agarose and covered with a coverslip, and observed under a fluoresence microscope (Olympus BH2). Images of *S. islandicus* cells were captured using a digital camera connected to the microscope.

### Western Blot and Hybridization

Cells were collected from 10 ml reference or drug-treated cultures and resuspended in 1× SDS loading buffer. The concentration of cell extracts was adjusted acoording to the A_600_ value of each cell sample to yield 1.3 × 10^7^ cells/μl, given a culture of A_600_ = 1.0 contains 1 × 10^9^ cells per ml. SDS-PAGE was conducted with 15% gel and proteins fractionated on each gel were transferred onto a PVDF membrane (Bio-Rad) by electronic transfer Trans-Blot SD Semi-Dry Transfer Cell (Bio-Rad). The membrane was first incubated with one of the primary rabbit antisera raised against RG1, Cren7, Alba, Sul7, Orc1-1, Orc1-2, Orc1-3, or PCNA3. Then, the membrane was incubated with the secondary antibody (anti-rabbit HRP, Thermo Fisher Scientific). After removing the unspecific binding, the second antiserum was detected using the ECL western blot substrate (Thermo Fisher Scientific). Hybridization signals were recorded by exposure of the membrane to an X-ray film (Agfa HealthCare, Belgium). Rabbit antiserum against RG1 (also name TopR1, SiRe_1581) was prepared in this work (raised with purified recombinant RG1 protein as the antigen in Innovagen, Sweden) whereas other antisera (against Cren7, Alba, Sul7, Orc1-1, Orc1-2, Orc1-3, or PCNA3) were reported to specifically detect the correponding proteins (Guo et al., 2003, 2008; Samson et al., 2013).

### Proteolysis of Sul7 and Cren7 in Cell Extract

Cells were collected from 50 ml treated or untreated *S. islandicus* culture by centrifugation, the cell pellet was washed once with the PBS buffer (pH 6.8) and resusepended in 400 μl of the same buffer. The cell suspension was sonicated to disrupt the cell envelope, and cell debris was removed by centrifugation, yielding cell extracts for proteolytic assay. Protein concentration in the cell extracts was determined by a BCA protein quantification kit (Thermo Fisher Scientific) and adjusted to 2.0 mg/ml. Two sets of aliquots were prepared for these cell extract samples, and one of them was subjected to EDTA treatment to inhibit metal-dependent protease activity (as references). These aliquots were incubated at 75°C for the timeframes indicated in each experiment and the reaction was terminated by adding SDS loading buffer. Residual contents of chromatin proteins Sul7 and Cren7 were analyzed by western blots with PCNA or RG1 as the reference. A relatively long exposure was used in some western experiments to ensure the detection of residual Cren7 and Sul7 proteins in the samples.

## Results

### Various DNA Damage Agents Induced DNA-Less Cell Formation in *S. islandicus*

**FIGURE 1:**
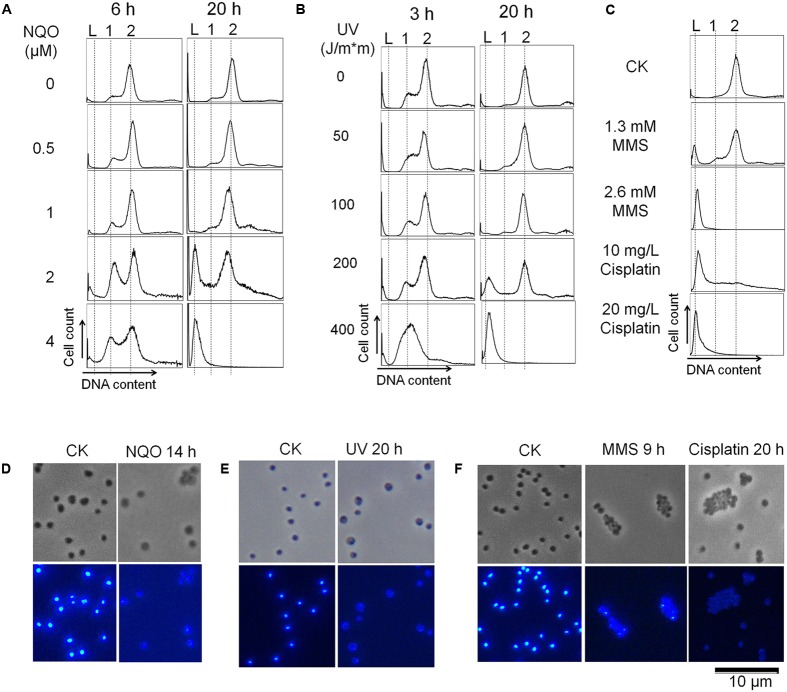
DNA damage agents induce DNA-less cell formation in *Sulfolobus islandicus.* **(A–C)** Exponentially growing cultures of the genetic host E233S1 were treated with 4-nitroquinoline 1-oxide (NQO), UV, MMS, and cisplatin with indicated dosages, and then analyzed by flow cytometry. The sampling time was indicated for NQO and UV treatment, while MMS- and cisplatin-treated cultures were analyzed at 20 h during treatment. Horizontal axis (DNA content) and vertical axis (cell count) are shown in liner scale. The DNA-less cells and cells containing one or two copies of genomic DNA are indicated with “L”, “1” and “2”, respectively. The data are the representatives of three independent experiments. **(D–F)** The cells treated with 4 μM NQO **(D)**, 400 J/m^2^ UV **(E)**, 2.6 mM MMS and 20 mg/L cisplatin **(F)** were analyzed with DAPI-staining and fluorescence microscopy. The sampling time was indicated. Upper panels: phase contrast; lower panels: DAPI.

Four different DNA damage agents were employed to investigate DNA damage-induced cell death in *S. islandicus* REY15A, including UV irradiation, MMS, cisplatin, and NQO. Exponentially growing *S. islandicus* cultures were treated with each DNA damage agent in different doses and grown as described in Section “Materials and Methods.” Cell samples were taken at the indicated time points during incubation and analyzed for cell viability and DNA content distributions. The results showed that all these DNA damage agents killed most cells in treated cultures (≥99.9%) at a lethal dosage, which were 400 J/m^2^ of UV irradiation, 4 μM NQO, 2.6 mM MMS and 20 mg/L cisplatin, respectively (Supplementary Figure 1). Flow cytometric analysis of these samples showed that each DNA damage agent induced DNA-less cell formation for almost all the cells in the treated culture (**Figures 1A–C** and **Table 1**). Furthermore, lowering doses of each DNA damage agent resulted in more survived cells as evidenced by increase of cell viability and decrease in DNA-less cell population in the treated cultures (**Figures 1A–C** and **Table 1**). Studying cellular DNA content by DAPI (4′,6-diamidino-2-phenylindole) staining and fluorescence microscopy revealed that, whereas all cells in the reference culture showed a strong staining by DAPI, little residual or no fluorescent signal was detected in the cells treated with the lethal dosages of each DNA damage agent (**Figures 1D–F**), consistent with the corresponding data flow cytometric data shown in **Figures 1A–C**.

**Table 1:** Percentage of DNA-less cells in the cultures after treatment of UV, NQO, MMS and cisplatin.

	UV (J/m^2^)		NQO (μM)		MMS (mM)		Cisplatin (mg/L)	
Dosages	200	400	2	4	1.3	2.6	10	20
Percentage of DNA-less cells (%)	24.8	96.4	30.7	96.5	15.1	97.7	55.2	97.8
*SD*	3.8	1.2	5.9	2.5	3.2	1.9	7.4	1.7

*The table is related to **Figures 1A–C**. Means and standard deviations (SD) are from three replicates*.

### Transient NQO Treatment Was Capable of Inducing DNA-Less Cell Formation

**FIGURE 2:**
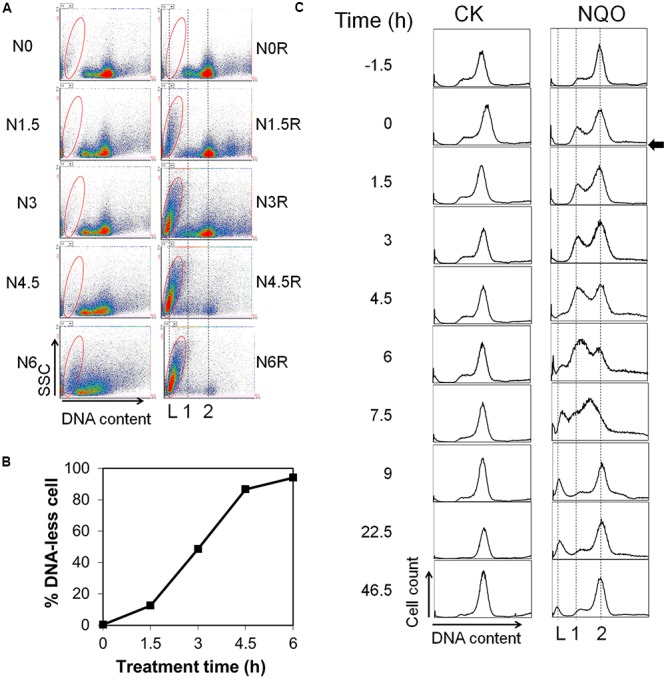
Analysis of the effects of transient NQO treatment on *Sulfolobus* cells. **(A)** *S. islandicus* cells were treated with 4 μM NQO for 0, 1.5, 3, 4.5, and 6 h respectively and analyzed by flow cytometry (N0, N1, N1.5, N3, N4.5, N6, left panels). Meanwhile, the cells were released and recovered in fresh medium for 24 h, when DNA content distributions were also analyzed by flow cytometry (N0R, N1R, N1.5R, N3R, N4.5R, N6R, right panels). The results were shown in FL2 (DNA content, horizontal axis)-SSC (side scattered light, vertical axis) cytograms. Both axes are shown in liner scale. **(B)** DNA-less cells from **(A)** were quantified. Very similar data were obtained from three independent experiments, and one set of representative data is shown. **(C)** Control and NQO-treated cells were released into fresh medium after incubation for 1.5 h (indicated as time “0” by an arrowhead). The cultures were further grown for 46.5 h, during which cell samples were taken DNA content profiles analyses. Populations of DNA-less cells (L), cells carrying one or two copies of chromosomes (1 and 2, respectively) are indicated.

To gain an insight into the DNA-less cell formation mediated by DNA damage agents, *S. islandicus* cultures were treated with 4 μM NQO for different time periods including 0, 1.5, 3, 4.5, and 6 h (N0, N1.5, N3, N4.5, and N6). Then the cells were collected after the treatments by centrifugation and resuspended in a fresh medium without NQO (N0R, N1.5R, N3R, N4.5R, or N6R). These released cultures were incubated for additional 24 h, and cell samples were taken for flow cytometry analysis. The data are displayed with DNA content (horizontal axis)-SSC (side-scattered light, vertical axis) cell-density plots (**Figure 2A**) and the DNA-less cells (enclosed with red circles) in each cytogram were quantified and the results show that 1.5, 3, and 4.5 h NQO treatment hardly induced any DNA-less cells, while 6 h NQO treatment led to about 10% DNA-less cells (**Figure 2A**, left panels). However, after released into fresh medium and recovery for 24 h, DNA-less cells were observed in each release experiment (**Figure 2A**, right panels), and the population of DNA-less cells was proportional to the time of NQO exposure (**Figure 2B**), indicating that transient NQO treatment is capable to induce DNA-less cells in the absence of the drug.

To yield a further insight into the cell death process, the DNA content distributions of N1.5R culture was followed during 46 h incubation. The analysis revealed three interesting features in cellular DNA content change during incubation: while cells with DNA content corresponding to G1 and S phase cells were accumulated for the first 6 h, their cellular DNA content increased in the following 3 h (from 6 to 9 h), and in addition, a small fraction of cells formed DNA-less cells in the same time period (**Figure 2C**). These findings suggest that transient NQO treatment induces cell cycle arrest in G1 and S phase as for UV irradiation (Frols et al., 2007; Gotz et al., 2007) not only during treatment but also after drug removal and that transient NQO treatment also yields irreversible DNA damage in a subset of cells in the culture, leading to cell death. Consistent with the former, we observed down-regulation of Orc1-1 and Orc1-3, two DNA replication initiators (Samson et al., 2013), and up-regulation of Orc1-2, a DNA damage-inducible protein (Gotz et al., 2007), during NQO treatment (Supplementary Figure 2). These data suggest that NQO-induced lesions exert similar effects on cell cycle in *Sulfolobus* as for UV radiation.

### Chromosomal DNA Degradation Was Accompanied by Degradation of Chromatin Proteins

**FIGURE 3:**
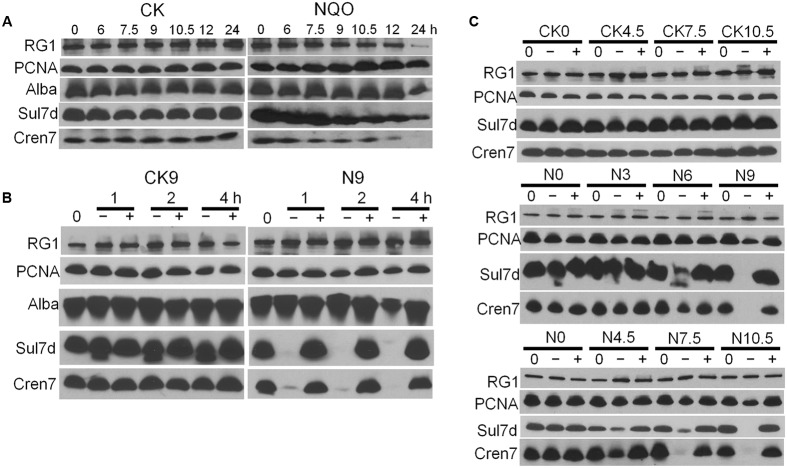
4-Nitroquinoline 1-oxide treatment activates a metalloprotease that specifically degrades Cren7 and Sul7. **(A)** The samples were taken at indicated time points from untreated or NQO-treated cultures and analyzed by western blot using corresponding antisera. **(B)** Cell extracts were prepared from untreated cells (CK) and the cells treated with NQO for 9 h (N9). The cell extracts were then incubated at 75°C with (+) or without EDTA (–) for 1, 2, and 4 h. “0” represents original cell extracts without incubation. At last, levels of indicated proteins were analyzed by western blot. **(C)** Cell extracts were prepared at indicated time points from untreated or NQO-treated cultures. Then, the degradation of Sul7 and Cren7 was analyzed by the *in vitro* proteolytic assay as described in **(B)**. Here, the cell extracts were incubated at 75°C for 2 h. In **(A)**, total protein from about 1.3 × 10^8^ cells, given that a culture of A_600_ = 1.0 contains 1 × 10^9^ cells per ml, was loaded onto the gel, while in **(B,C)**, about 20 μg total protein was analyzed.

Previous studies suggest that several unique chromatin proteins, Cren7, Sul7 and Alba, could function in protecting genome integrity in *Sulfolobus* (Guo et al., 2008; Laurens et al., 2012; Driessen et al., 2013). Here, the level of chromatin proteins in NQO-treated cells was analyzed by immunoblotting. We found that the levels of Cren7 and Sul7 started to decrease at 7.5 and 9 h after the addition of NQO, respectively (**Figure 3A**). These results are consistent with the observation that the reduction of cellular DNA content occurred at 6 to 12 h (Supplementary Figure 3), suggesting that the reduction in cellular chromatin protein content is correlated with the extent of chromosomal DNA degradation in this archaeon. We also analyzed the levels of Cren7 and Sul7 in the UV-, MMS- and cisplatin-treated cells, and the results indicated that chromatin protein degradation occurred during each DNA damage treatment at a lethal dose (Supplementary Figure 4). Therefore, chromatin protein degradation probably represents a general feature upon lethal DNA damage in *S. islandicus*.

To learn how the chromatin proteins were depleted from the NQO-treated cells, we employed an *in vitro* proteolytic assay to detect chromatin degradation in cell extracts. Two cell extract samples were prepared: one was the cell sample withdrawn at 9 h after NQO supplementation (N9) while the other was the corresponding untreated control cells (CK9). Each cell extract sample was incubated at 75°C for the indicated time points, and analyzed by immunoblotting with antibodies against the chromatin proteins including Alba, Cren7 and Sul7, with antisera against reverse gyrase 1 (RG1) and the sliding clamp protein PCNA3 as references. To ensure the detection of possible residual Cren7 and Sul7, the blot has been exposed to X-ray films for a relatively long time. As shown in **Figure 3B**, whereas Sul7 and Cren7 dropped to an undetectable level in N9 sample after 1 h incubation, the contents of Alba, RG1 and PCNA3 were almost the same (**Figure 3B**). In comparison, little proteolysis of all tested proteins was observed in the control sample (**Figure 3B**). Furthermore, the addition of EDTA completely abolished proteolytic degradation of the chromatin proteins (**Figure 3B**), indicating that the proteolysis is metal ion-dependent.

To test whether the chromatin proteins were targeted for degradation via substrate modification, the reference sample (no drug treatment, CK9) was mixed with the N9 sample at a 1:1 ratio (CK9:N9), and the proteolytic degradation of Cren7 and Sul7d in the mixture was analyzed. We found that chromatin proteins derived from both samples were hydrolyzed by the protease present in the N9 sample (Supplementary Figure 5A) and, moreover, the time required for the digestion of Sul7 was proportional to the fold of N9 dilution (Supplementary Figure 5B). This indicated that proteolytic degradation was equally efficient for chromatin proteins of the NQO-treated cells and those of untreated cells.

Next, the *in vitro* proteolytic assay was employed to reveal the onset of protease activity during NQO treatment. Cell extracts were prepared from cell samples collected at a series of time points during NQO-treatment, and residual contents of Cren7 and Sul7 in the cell extracts were analyzed after incubation. The results revealed that degradation of Sul7 and Cren7 occurred at 4.5 h after NQO treatment and an increased level of the proteolytic activity was observed in each of the following cell samples including N6, N7.5, N9, and N10.5 samples (**Figure 3C**). These data indicated that protease activation occurred, either earlier than, or simultaneously with, genomic DNA degradation and chromatin protein degradation. Furthermore, SDS-PAGE of samples of the protease assay detected the specific depletion of the <10 kDa protein band (corresponding to the chromatin proteins) from the total protein (Supplementary Figure 6). These results indicated that Cren7 and Sul7 were specifically degraded by an unknown protease that was activated by NQO treatment.

### Membrane Depolarization Preceded Genomic DNA Degradation

To gain more insights into cellular changes in *S. islandicus* cells during NQO treatment, two cell-staining methods were employed to evaluate the membrane potential and the integrity of cell membrane in the NQO-treated cells. These included DiBAC_4_ (*bis*-1,3-dibutylbarbituric acid trimethine oxonol) and the dye mix of SYTO9 and PI. DiBAC_4_ has a high affinity for the depolarized membrane of bacterial and eukaryotic cells and increased DiBAC_4_ signal can be used as an indicator of cell membrane depolarization (Bortner et al., 2001; Erental et al., 2012). While SYTO9 is able to enter live *Sulfolobus* cells with intact membrane and stains them green, PI only enters dead cells that have lost membrane integrity and stains them red (Leuko et al., 2004; Bize et al., 2009). The same set of untreated or NQO-treated cell samples were stained by three different methods, i.e., EB/mithramycin, DiBAC_4_ and SYTO9/PI, and analyzed by flow cytometry to reveal DNA-less cells, cells with depolarized membrane (DiBAC-positive) and cells that lost the membrane integrity (PI-positive), respectively.

**FIGURE 4:**
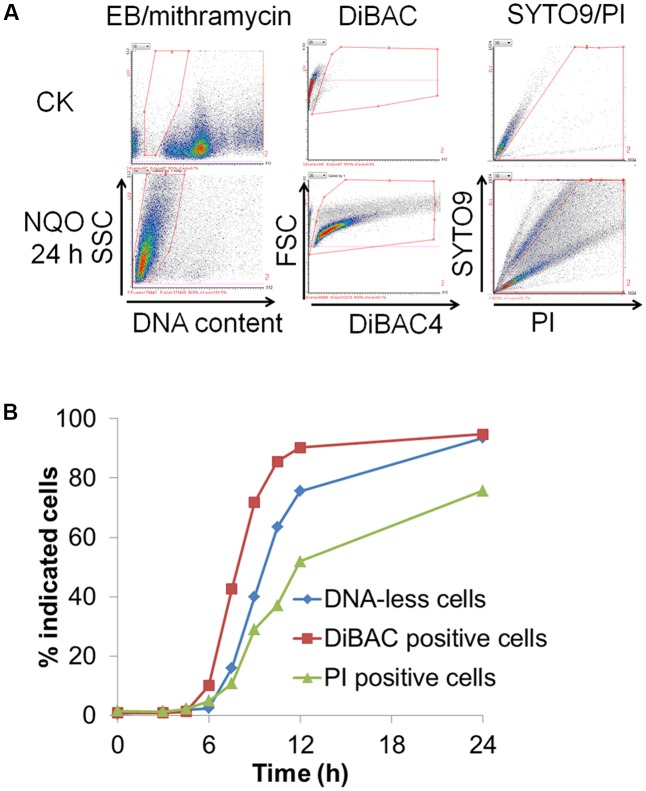
Membrane depolarization occurs prior to chromosome degradation and loss of membrane integrity during the lethal NQO treatment. **(A)** Untreated and NQO-treated (24 h) cells were stained with DiBAC4, EB/mithramycinA and SYTO9/PI, respectively. The cells for EB/mithramycinA staining were prefixed by 70% ethanol for overnight. Then, the samples were analyzed by flow cytometry and the results were shown in FL2 (DNA content, horizontal axis)-SSC (vertical axis), FL1 (DiBAC, horizontal axis)-FSC (forward scattered light, vertical axis) and FL2 (PI, horizontal axis)-FL1 (SYTO9, vertical axis) cytograms, respectively. In the experiments, DiBAC-stained cells and SYTO9-stained cells emit green fluorescence (FL1), while PI-stained cells emit red fluorescence (FL2). Both axes are shown in liner scale. The entangled area indicates DNA-less cells (left panels), DiBAC-positive cells (middle panels) and PI-positive cells (right panels). **(B)** Quantification of the percentage of DNA-less cells, DiBAC-positive cells and PI-positive cells during NQO treatment. The data are the representatives of three independent experiments.

As exemplified with the sample of 24 h NQO treatment in **Figure 4A**, whereas the reference culture contains essentially no DNA-less, DiBAC4-positive or PI-positive cells (entangled area), a large number of such cells were observed in the NQO-treated culture (entangled area). Further, the percentages of the three types of cells, i.e., DiBAC4-positive, PI-positive and DNA-less cells, were calculated with the ratio of entangled cells to total cells and the resulting data were plotted against the incubation time (Supplementary Figure 7). As shown in **Figure 4B**, DiBAC-positive cells always outnumbered DNA-less cells, whose number was in turn larger than that of PI-positive cells. In addition, kinetic study on the formation of the three distinct types of cells with lower doses of NQO (2, 3, or 3.5 μM) revealed similar kinetics for these NQO treatments although different fractions of cells were recovered from NQO-mediated damage and resumed cell growth in the cultures treated with lower dosages of NQO (Supplementary Figure 8A). Further, similar phenomena were also observed during lethal MMS treatment (Supplementary Figure 8B). Therefore, we concluded that the loss of membrane potential, DNA-less cell formation and the loss of membrane integrity occurred sequentially in the cell death process.

### Excess Proton Influx Induced Chromosomal DNA Degradation

**FIGURE 5:**
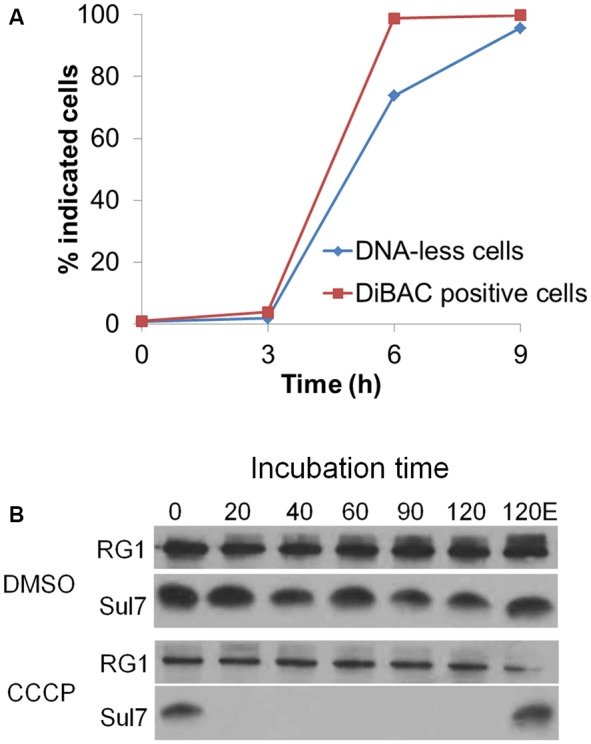
Proton uncoupler mediated membrane depolarization and chromosome degradation. **(A)** The cells treated with CCCP were taken at indicated time points, stained with DiBAC4 and EB/mithramycinA, respectively, and analyzed with flow cytometry. The percentage of DNA-less cells and DiBAC-positive cells was quantified. The data are the representatives of three independent experiments. **(B)** The cell extracts were prepared after 6 h DMSO or CCCP treatment. Then, the cell extracts were incubated at 75°C for 120 min, during which levels of Sul7 from 20 μg of total protein was monitored by western blot. “120E” represents the sample supplemented with EDTA and incubated at 75°C for 120 min.

The sequential occurrence of membrane depolarization and chromosomal DNA degradation suggested that the change in membrane potential could play an important role in the NQO-induced cell death. *Sulfolobus* cells possess a small inside negative membrane potential that directs proton influx through A_0_A_1_-ATPase to synthesize ATP from ADP, taking the advantage of the pH gradient across membrane (Schafer, 1996; Steinert et al., 1997). It has been reported that proton gradient uncouplers induce proton influx and abolish the negative membrane potential (Lubben and Schafer, 1989). Here, we employed CCCP, a proton gradient uncoupler that induces apoptosis in eukaryotes (Shimizu et al., 1998; Yang et al., 2001) to investigate its effect on the growth of *S. islandicus*. The results showed that CCCP induced membrane depolarization and chromosome degradation in *S. islandicus* at 6 h of drug treatment (**Figure 5A** and Supplementary Figure 9) and that the chromatin-degrading protease was very active in the CCCP-treated cells (**Figure 5B**).

### NQO-Induced Chromosomal Degradation Was Dependent on A_0_A_1_-ATPase

**FIGURE 6:**
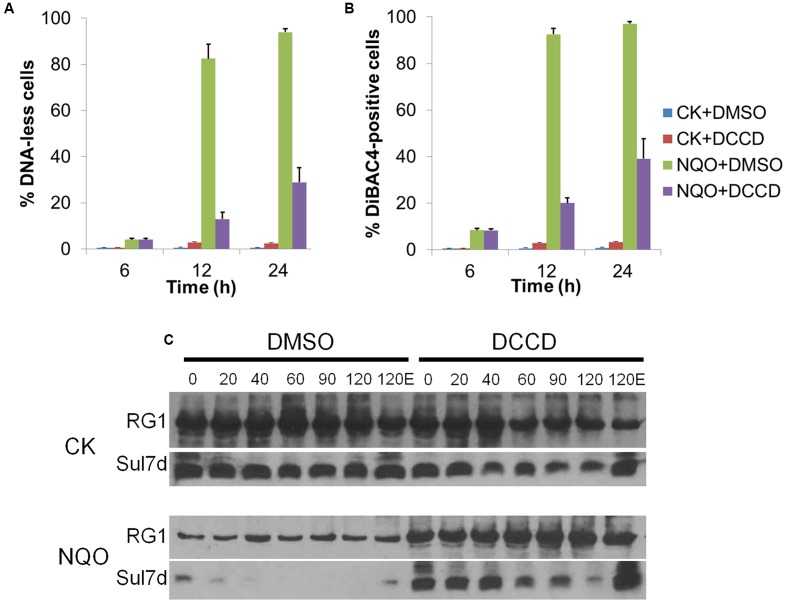
Dicyclohexylcarbodiimide (DCCD) inhibits the NQO-induced DNA-less cell formation and proteolytic degradation of chromatin protein. **(A,B)** The cultures after 6 h NQO treatment or no treatment were supplemented with DMSO or DCCD, respectively. Then, at 12 and 24 h after NQO supplementation, the percentage of DNA-less cells **(A)** and DiBAC-positive cells **(B)** was analyzed by flow cytometry. Error bars represent SD of three independent experiments. **(C)** Cell extracts were prepared at 12 h during the treatments as described in **(A,B)**. Then, the degradation of Sul7 was analyzed by the *in vitro* proteolytic assay as described in **Figure 3B**. The cell extracts were incubated at 75°C for 120 min, during which levels of Sul7 from 20 μg of total protein was monitored by western blot. “120E” represents the sample supplemented with EDTA and incubated for 120 min.

In the absence of proton uncouplers, the membrane of *Sulfolobus* cells is highly impermeable for proton, and the ATP synthase, A_0_A_1_-ATPase, is the main channel for proton influx (Baker-Austin and Dopson, 2007). To investigate whether NQO induces chromosomal degradation via the A_0_A_1_-ATPase-mediated proton influx, we analyzed the effect of DCCD, a chemical that binds A_0_A_1_-ATPase and inhibits both proton influx and ATP synthesis in *S. acidocaldarius* (Lubben and Schafer, 1989), on NQO-induced cellular changes. The results showed that when supplemented into the cultures treated with NQO for 6 h, DCCD strongly impaired NQO-induced membrane depolarization and DNA-less cell formation at both 12 and 24 h after NQO addition (**Figures 6A,B** and Supplementary Figure 10). Further, in the cells treated with NQO for 12 h, the chromatin-degrading protease was very active, degrading most Sul7 after 20 min incubation, while in the NQO-treated cells supplemented with DCCD at 6 h, the activity of the chromatin-degrading protease was suppressed (**Figure 6C**), suggesting that DCCD inhibits NQO-induced chromosome degradation. Further, the analysis of the effect of DCCD on CCCP-induced cellular changes showed that DCCD could hardly affect CCCP-induced membrane depolarization and chromosomal DNA degradation (Supplementary Figure 11). Together, these data suggest that the A_0_A_1_-ATPase is involved in membrane depolarization and chromosomal DNA degradation in the NQO-treated cells.

## Discussion

### NQO-Induced DNA Damages Mediate DNA Replication Stresses Similar to Those Reported for UV Irradiation in *S. islandicus*

Among the four DNA damage agents studied here, three of them have been shown to cause DNA damage in organisms of *Sulfolobus* genus, including UV lights, MMS and cisplatin (Reilly and Grogan, 2002; Valenti et al., 2006; Frols et al., 2007; Gotz et al., 2007), but whether NQO also exhibits DNA damage in an archaeon was not investigated before. It has been reported that NQO functions as a DNA damage agent in bacteria and eukaryotes (Ikenaga et al., 1977; Williams et al., 2010), and the drug relies on cellular metabolic activities to form stable bulky quinolone adducts on bases of DNA (Bailleul et al., 1989). Further, genetic studies in *Escherichia coli* show that the genes required for UV resistance are also essential for mediating NQO resistance, suggesting that NQO-induced DNA lesions are to be repaired by NER (Williams et al., 2010). If left unrepaired by the time of DNA replication, the large quinolone adducts would block DNA synthesis, leading to stalled replication forks. Our researches on the effects of NQO on cell cycle in *S. islandicus* cells show that transient NQO treatments cause accumulation of the cell population showing a DNA content corresponding to G1/S cells, suggesting that NQO-mediated DNA lesions suppress DNA replication elongation and/or inhibit DNA replication initiation. The latter is supported by the data that NQO induces down-regulation of Orc1-1 and Orc1-3. In summary, NQO affects cell cycle in *S. islandicus* in the same fashion as observed for UV treatment in *Sulfolobus* (Frols et al., 2007; Gotz et al., 2007), indicating that NQO functions as a DNA damage agent that mimics the DNA damage effect of UV radiation in *Sulfolobus*.

### DNA-Less Cell Formation Represents a Common Outcome in Response to Various DNA Damage Agents in *S. islandicus*

To date, DNA-less cell formation has only been reported for *Sulfolobus* species, but the phenomenon has been observed in several different experiments. First, investigation of flow cytometry profiles of *S. acidocaldarius* cultures treated with actinomycin D, a transcription inhibitor that blocks replication elongation has led to the discovery of DNA-less cell formation in this archaeon (Hjort and Bernander, 2001). Second, examination of the fate of ultra-violet (UV)-irradiated *S. acidocaldarius* and *S. solfataricus* cells has detected a large number of DNA-less cells (Gotz et al., 2007) upon UV radiation. Here, we demonstrate that different DNA damage agents can induce DNA-less cells in *S. islandicus*, indicating that DNA-less cell formation is common consequence in response to different DNA damage agents.

### DNA-Less Cell Formation Accompanies Degradation of Crenarchaeal Chromatin Proteins

While euryarchaea code for histone-like proteins that form nucleosome-like structures to maintain genome integrity, crenarchaea do not encode any histone-like protein (Driessen et al., 2013). Instead, the latter encode crenarchaea-specific chromatin proteins that are implicated in protecting cellular DNA. Here, we show that chromosomal DNA degradation is accompanied by degradation of Cren7 and Sul7 chromatin proteins during DNA-less cell formation in *S. islandicus*. It has been shown that chromosomal DNA degradation is concomitant with RG1 degradation during MMS treatment (Valenti et al., 2006). More recently, we report that MMS-induced DNA-less cell formation occurs after RG1 degradation but is coincided with chromatin protein degradation (Han et al., 2017). Although RG1 reduction is also observed in NQO-treated cells, we do not attribute that to NQO treatment because the event occurs only at a late stage in NQO treatment (24 h) when cell density by A_600_ value decreases. Further, RG1 degradation in the NQO-treated cell extract was not detected, in agreement with the observation that RG1 is not degraded in *S. solfataricus* upon UV treatment (Napoli et al., 2004). Therefore, our results support the conclusion by Valenti et al. (2006) that RG1 degradation is a specific cellular response to MMS treatment. Nevertheless, for all three DNA damage treatment, chromatin protein degradation is correlated to DNA-less cell formation, reinforcing the conclusion that crenarchaeal chromatin proteins protect genomic DNA *in vivo*.

Another important question raised from our work is whether chromosomal DNA degradation in *Sulfolobus* involves the activation of an unknown nuclease. It has been shown that DNA is not stable at high temperatures in which non-enzymatic DNA degradation occurs spontaneously (Lindahl, 1993). Since the binding of chromatin proteins to DNA *in vitro* greatly increases its thermal stability (Guo et al., 2008), it has been reasoned that these proteins and DNA form chromatin-like structures *in vivo* to facilitate genome integrity in crenarchaea. It is thus plausible that protein depletion from crenarchaeal chromatins would be sufficient to facilitate spontaneous chromosomal DNA degradation. Nevertheless, whether enzymatic degradation is involved in NQO-induced DNA degradation remains to be investigated.

We have noticed an apparent inconsistence between the immunoblotting data of total protein and those obtained from *in vitro* protease assay of the same cell extract samples. This difference probably reflects the difference in substrate accessibility in the two assays: whereas cell compartmentation renders it impossible for the protease in one cell to access its substrates in any other cells, this barrier no longer exists in cell extracts. Therefore, when the protease is activated in the subpopulation of cells that have initiated the cell death process, the enzyme can only degrade chromatin proteins within the cell; once cell extracts are prepared, chromatin proteins in the cell extracts are equally accessible to the protease and are readily degradable by the enzyme. This has been further demonstrated by comparing the activities present in mixing cell extracts of the NQO-treated cell extract and those of untreated control shown in Supplementary Figure 5.

The NQO-activated archaeal metalloprotease remains to be identified thus far. Several proteases that function in regulating cell death have been characterized in Eukaryotes and Bacteria. The former class is composed of a special class of proteases named caspases that are sequentially activated in apoptosis (Riedl and Shi, 2004). In the latter class, diverse proteases mediate cell death by specific degradation of different antitoxins in bacteria (Hazan et al., 2004; Engelberg-Kulka et al., 2006). Nevertheless, homologs of caspase have not been identified in archaea thus far and the bacterial proteases do not affect chromosomal structure and neither do they induce DNA degradation (Dwyer et al., 2012; Erental et al., 2012). It is thus unlikely that any of these *Sulfolobus* proteases could be related to any of eukaryal caspases or bacterial antitoxin-degrading proteases. Plausibly, the proteases that degrade *Sulfolobus* DNA structural proteins represent a novel type of protease. In an early work, it has been shown that a metalloprotease is involved in specific degradation of RG1 in *S. solfataricus* (Valenti et al., 2006; Visone et al., 2014). To date, whether different proteases are responsible for degradation of Cren7, Sul7 and RG1 has not been addressed experimentally. Nevertheless, these proteases have been implicated in mediating genome instability in these organisms.

### A_0_A_1_-ATPase Functions in DNA-Less Cell Formation

A_0_A_1_-ATPase synthesizes ATP from ADP by the passive flux of protons through it in *Sulfolobus*, which is mainly dependent on the large pH gradient between the cytoplasm and the environment (Schafer, 1996; Steinert et al., 1997). In *Sulfolobus*, ATP synthesis can be inhibited by DCCD that inhibits proton influx through A_0_A_1_-ATPase, and proton uncouplers that mediate excess proton influx across membrane. However, we show that the two types of drugs exert different effects on DNA-less cell formation in *Sulfolobus*. Firstly, DCCD impairs NQO-induced DNA-less cell formation, suggesting a role of A_0_A_1_-ATPase in the process. However, CCCP, a proton uncoupler, induces DNA-less cell formation even in the presence of DCCD. CCCP is capable to drive protons across lipid bilayers, suggesting that an excess proton influx is sufficient to induce cell death in *Sulfolobus* featured with DNA-less cell formation. Together, the distinct effects of DCCD on NQO and CCCP-induced DNA-less cell formation suggest that proton influx mediated by A_0_A_1_-ATPase could be responsible for triggering the cell death observed in *Sulfolobus* during NQO treatment.

The importance of ATP synthase in cell death has been revealed in mitochondrion-dependent apoptosis in which inducing excess proton influx into mitochondria triggers apoptosis (Shimizu et al., 1998). In fact, the mitochondrial F_0_F_1_-ATPase has multiple functions in the programmed cell death. For example, the enzyme has an essential role in the apoptosis induced by the proapoptotic protein Bax (Matsuyama et al., 1998), and it is also involved in the generation of reactive oxygen species (ROS) and execution of cell death (Santamaria et al., 2006). Further investigation of A_0_A_1_-ATPase would reveal whether the archaeal enzyme also has multiple functions in the DNA-less cell formation as well as how the archaeal cell death mechanism is related to the eukaryotic apoptosis.

## Conclusion

Our study on the NQO-induced cell death in *S. islandicus* has revealed that the drug induces cell death primarily by producing DNA lesions to chromosomal DNA, and the cell death process is featured with following events, including membrane depolarization, proteolytic degradation of chromatin proteins and chromosomal DNA degradation. We also demonstrate that A_0_A_1_-ATPase has an essential function in the cell death process. Finally, we propose that the DNA-less cell formation can be regarded as an archaeal type of programmed cell death.

## Author Contributions

WH, YX, and QS designed experiments; WH, YX, and XF conducted the experiments; YL, LH, and YS contributed essential agents; WH and QS wrote the article. All authors participated in data discussion, commented on the manuscript.

## Conflict of Interest Statement

The authors declare that the research was conducted in the absence of any commercial or financial relationships that could be construed as a potential conflict of interest.
