# Supplementary material for: NQO-Induced DNA-Less Cell Formation Is Associated with Chromatin Protein Degradation and Dependent on A0A1-ATPase in Sulfolobus
Source: Front Microbiol. 2017 Aug 14;8:1480. doi: 10.3389/fmicb.2017.01480 (PMC5557786; doi:10.3389/fmicb.2017.01480)
Supplement: Supplementary file 1 [file Data_Sheet_1.DOCX]

Supplementary Material

NQO-induced DNA-less cell formation is associated with chromatin protein degradation and dependent on A_0_A_1_-ATPase in *Sulfolobus*

Wenyuan Han^1^, Yanqun Xu^1,2^, Xu Feng^1,2^, Yun Xiang Liang^2^, Li Huang^3^, Yulong Shen^4^ and Qunxin She^1,2*^

^1^Archaea Centre, Department of Biology, University of Copenhagen, Ole MaaløesVej 5, Copenhagen Biocenter, DK-2200 Copenhagen N, Denmark

^2^ State Key Laboratory of Agricultural Microbiology and College of Life Science and Technology, Huazhong Agricultural University, 430070 Wuhan, China

^3^ State Key Laboratory of Microbial Resources, Institute of Microbiology, Chinese Academy of Sciences, Beijing, China

^4^ State Key Laboratory of Microbial Technology, Shandong University, 27 Shanda Nan Rd., Jinan, 250100, P. R. China

^*^ Corresponding author: Tel. +45 3532 2013, Fax +45 3532 2128, email: qunxin@bio.ku.dk


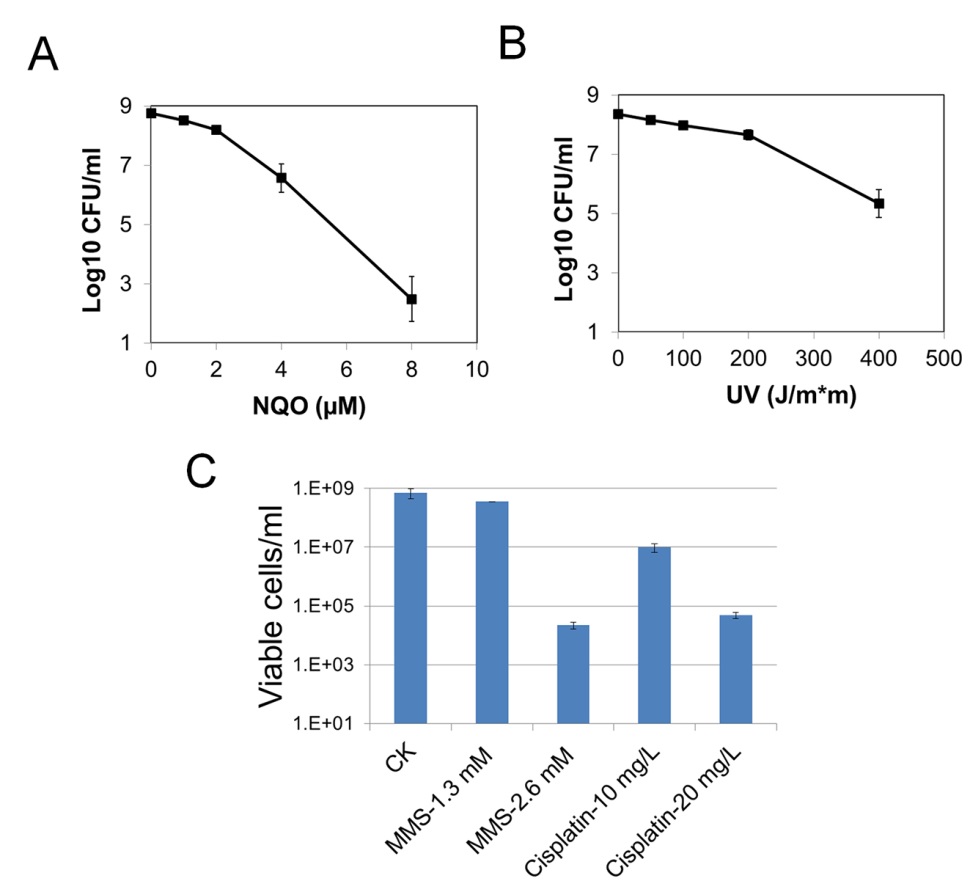


**Supplementary Fig. 1**.

Exponential growing cultures of *S. islandicus* were treated with NQO (A), UV (B), MMS and cisplatin (C) at indicated dosages. Cell viability was analyzed by colony formation assay at 20 h for NQO-, MMS- and cisplatin-treated cultures, or after 3 h’s recovery for UV-treated cultures. Error bars represent SD of three independent experiments.


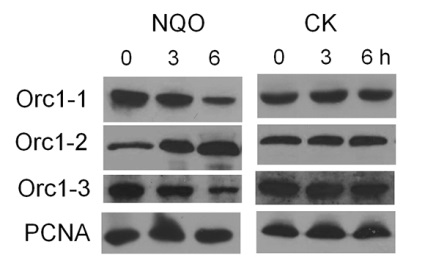


**Supplementary Fig. 2.** NQO treatment induces down-regulation of Orc1-1 and Orc1-3 and up-regulation of Orc1-2. Exponential phase *S. islandicus* cultures were treated with 4 µM NQO or not and at indicated time points, the levels of indicated proteins were analyzed with corresponding antisera.


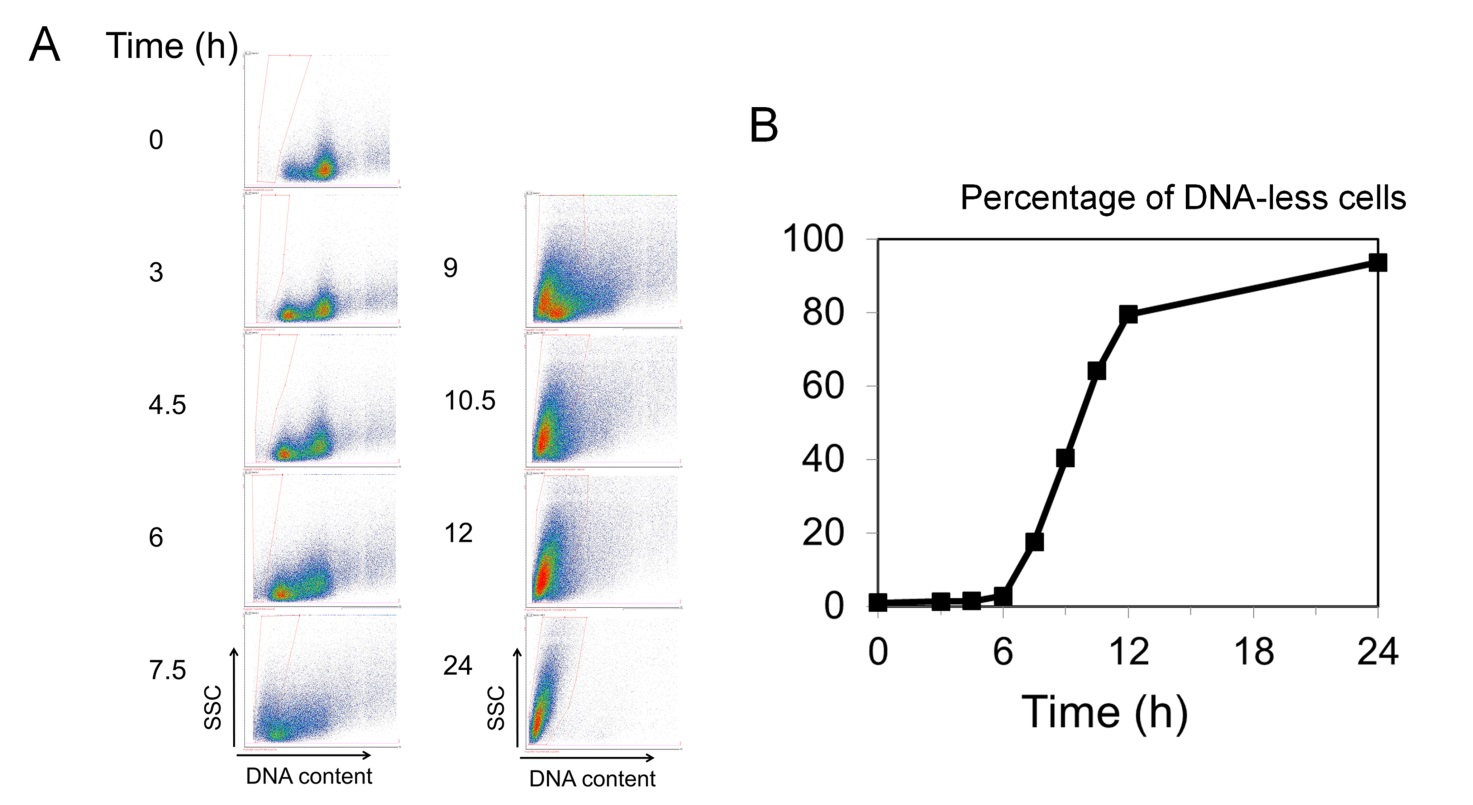


**Supplementary Fig. 3**. Flow cytometry analysis of NQO-induced DNA-less cell formation. This figure is related to Fig. 3A. (A) The NQO-treated samples were taken at indicated time points and analyzed with flow cytometry. The data were shown in DNA content-SSC cytograms. (B) The quantification of DNA-less cell formation during NQO treatment.


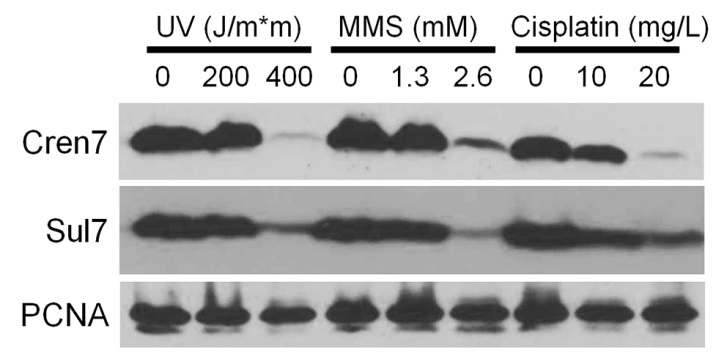


**Supplementary Fig. 4**. UV, MMS and cisplatin treatment induces chromatin protein degradation. Exponential phase *S. islandicus* cultures were treated with MMS and cisplatin at indicated dosages for 20 h or exposed to UV and grown for 20 h. Then the levels of Cren7, Sul7 and PCNA were estimated based on western blot hybridization using corresponding antisera.


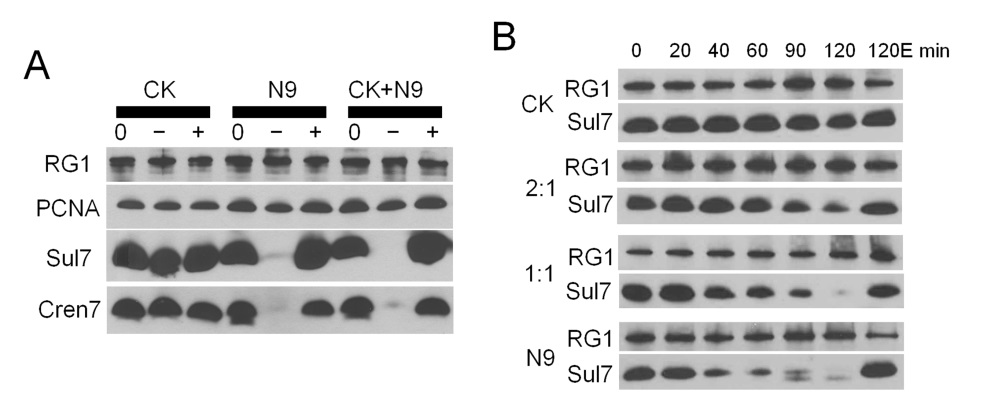


**Supplementary Fig. 5**. NQO-activated protease also degrades Sul7 and Cren7 derived from untreated cells.

(A) Different cell extracts (CK: cell extract of untreated cells, N9: cell extract of the cells after 9 h’s NQO treatment, CK+N9: the mixture of CK and N9 with 1:1 ratio) were incubated at 75 ^o^C for 4 h with EDTA (+) or without EDTA (−). “0” represented the samples without incubation. Then, the levels of indicated proteins were analyzed with western blot.

(B) CK and N9 cell extracts were mixed with 2:1 and 1:1 respectively. Both separate and mixed cell extracts were incubated at 75 ^o^C for 120 min and aliquots were taken at indicated time points for western blot analysis. In 120E, EDTA was added to 3 mM to inhibit the protease activity (negative control).


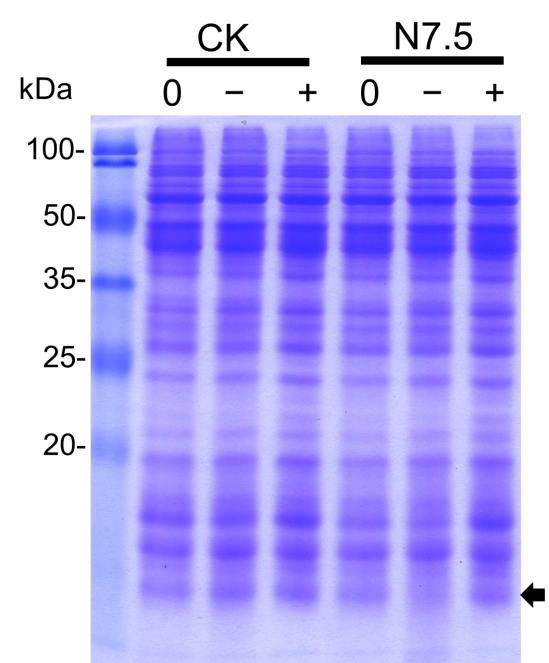


**Supplementary Fig. 6**. SDS-PAGE analysis of the degradation of chromatin proteins.

CK (cell extract of untreated cells) and N7.5 (cell extract of the cells after 7.5 h’s NQO treatment) were analyzed by the in vitro proteolytic assay as described as Fig. 3C. Then the samples were analyzed by SDS-PAGE. The arrow indicates the position of proteins corresponding to chromatin proteins.


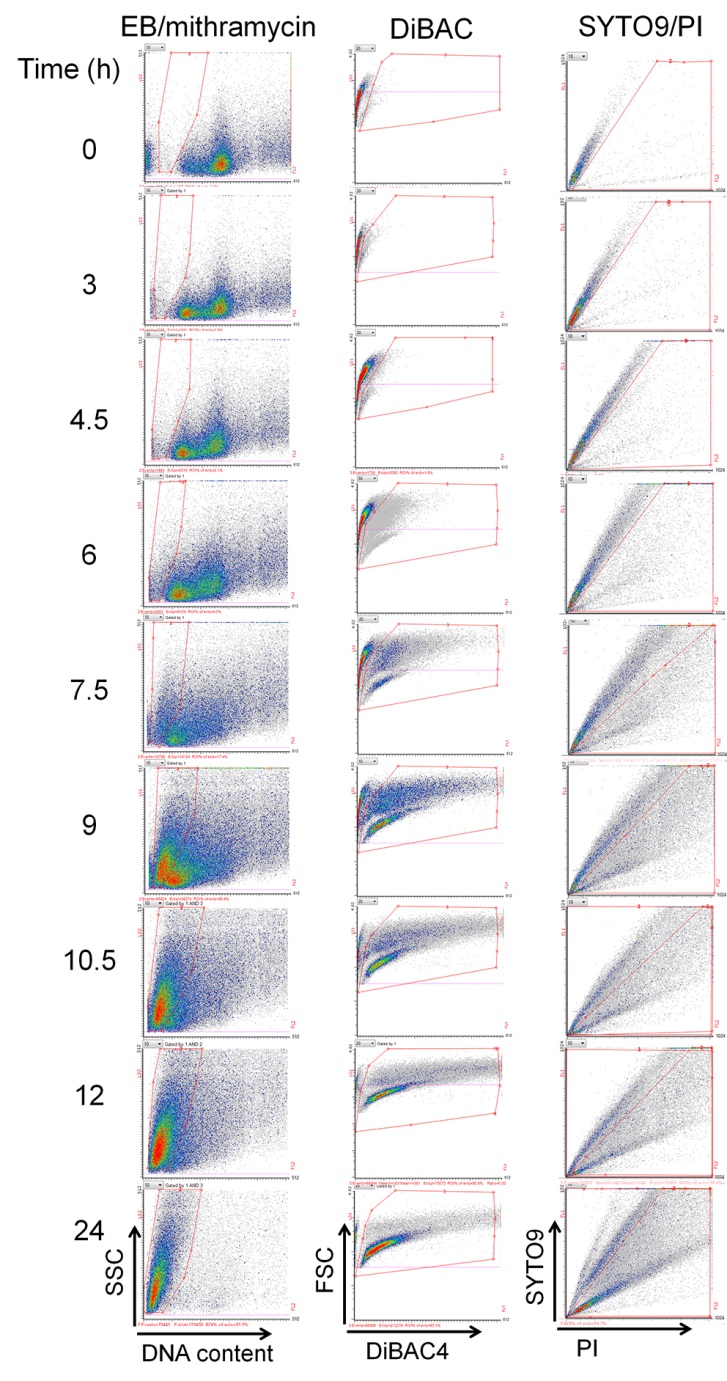


**Supplementary Fig. 7.** Kinetics of the formation of DNA-less, DiBAC-positive and PI-positive cells during NQO treatment. The NQO-treated cells were taken at indicated time points and stained with EB/mithramycinA, DiBAC4 and SYTO9/PI, respectively. Then, the samples were analyzed by flow cytometry and the results were shown in FL2 (DNA content)-SSC, FL1 (DiBAC)-FSC and FL2 (PI)-FL1 (SYTO9) cytograms, respectively. The figure is related to Figure 4B.


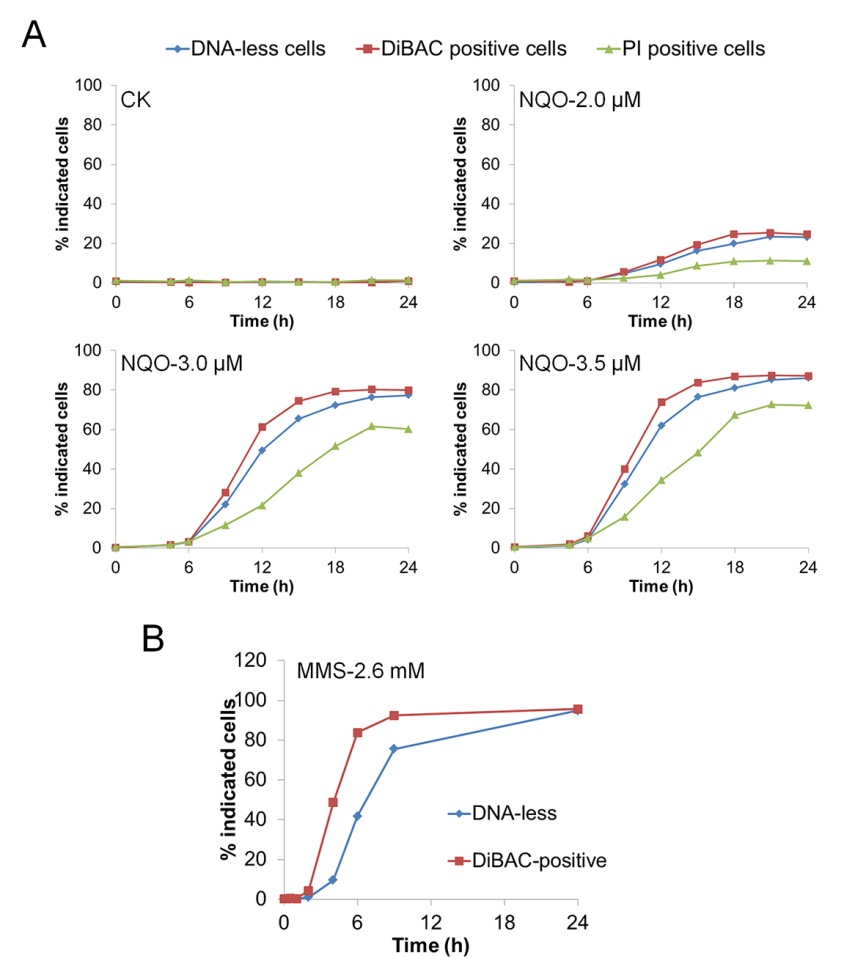


**Supplementary Fig. 8.** (A) Effects of different NQO concentrations on the formation of DNA-less cells, DiBAC-positive cells and PI-positive cells during NQO treatment. Exponential growing *S. islandicus* cultures were treated with NQO at indicated concentrations for 24 h, during which cell samples were withdrawn and flow cytometric analyses as described in experimental procedures. (B) Formation of DNA-less cells and DiBAC-positive cells during 2.6 mM treatment.


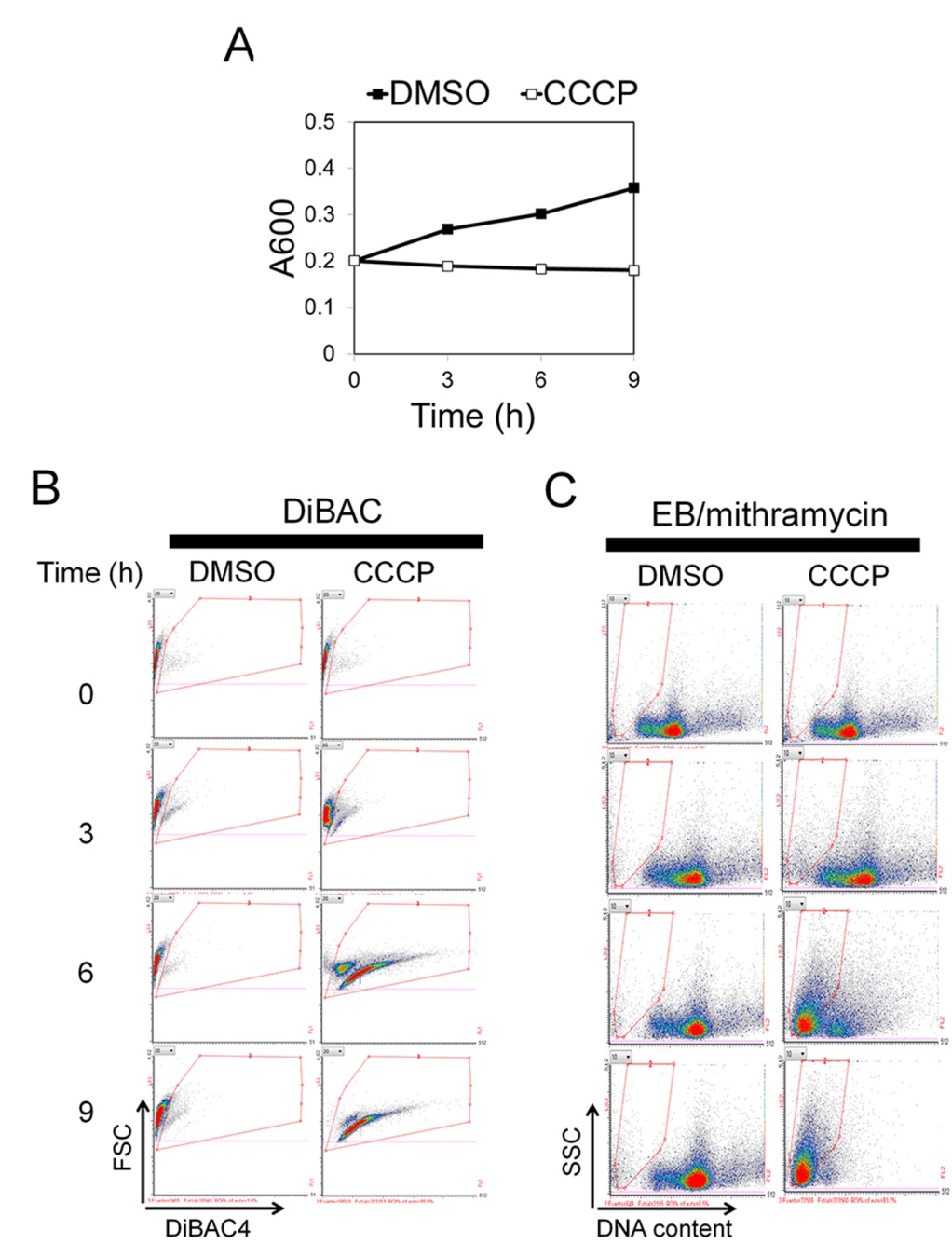


**Supplementary Fig. 9.** CCCP induces membrane depolarization and chromosome degradation.

(A) Growth curves of the cultures treated with CCCP and control (DMSO).

(B) and (C)The cells treated with DMSO and CCCP were taken at indicated time points and stained with DiBAC4 (B) and EB/mithramycinA (C), respectively. Then the samples were analyzed by flow cytometry and the results were shown in FL1 (DiBAC)-FSC (B) and FL2 (DNA content)-SSC (C) cytograms.


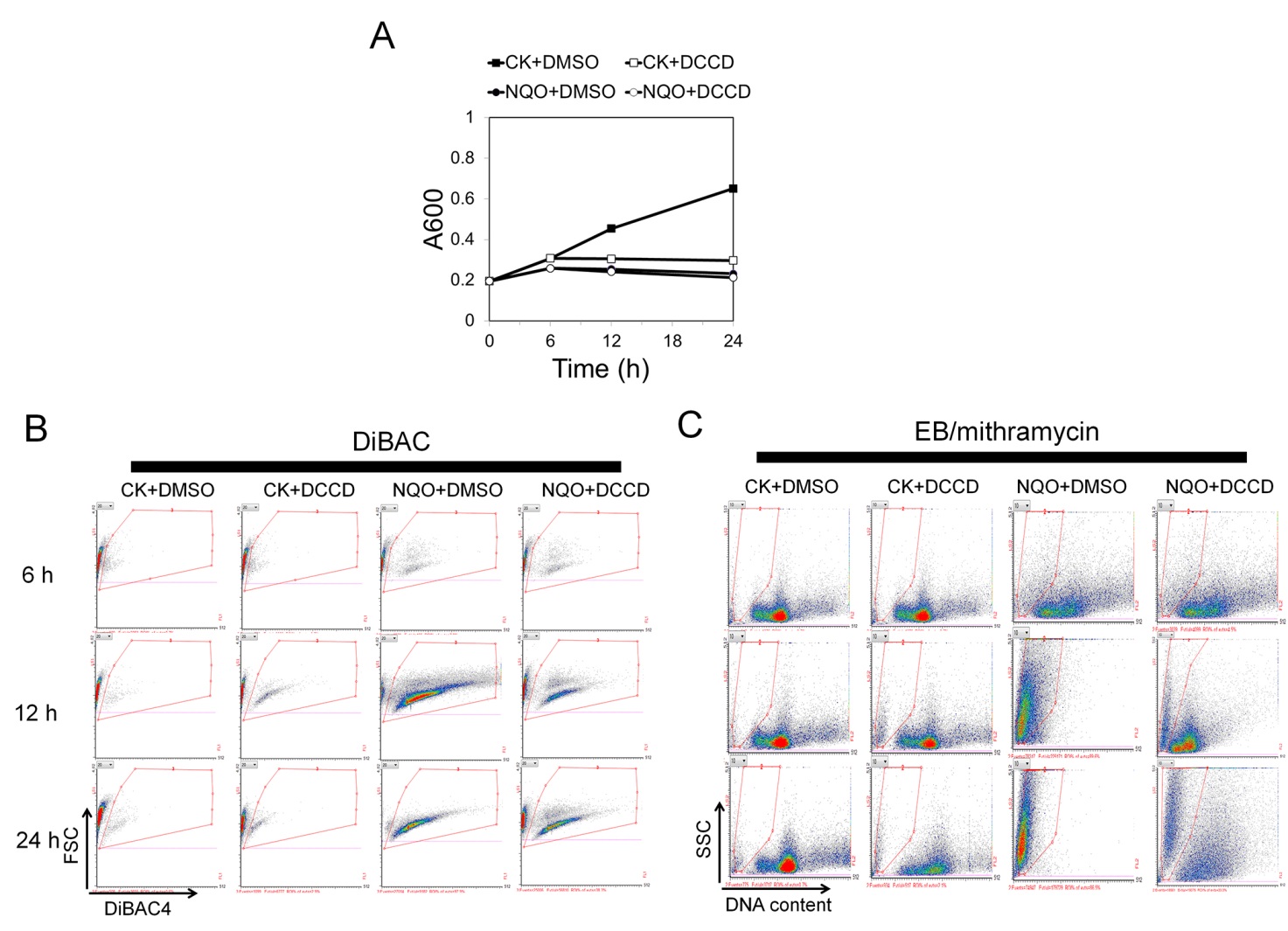


**Supplementary Fig. 10.** DCCD inhibits NQO-induced membrane depolarization and chromosome degradation.

(A) Exponential phase cultures were treated with NQO or grown (as CK) for 6 h, and then, cultures were supplemented with DMSO or DCCD, respectively. At 12 h and 24 h (6 h and 18 h after the addition of DMSO or DCCD), the optical density was measured.

(B) and (C) The sample from (A) were also analyzed by flow cytometry after stained by DiBAC (B) or EB/mithramycinA (C). The results were shown in FL1 (DiBAC)-FSC (B) and FL2 (DNA content)-SSC (C) cytograms.


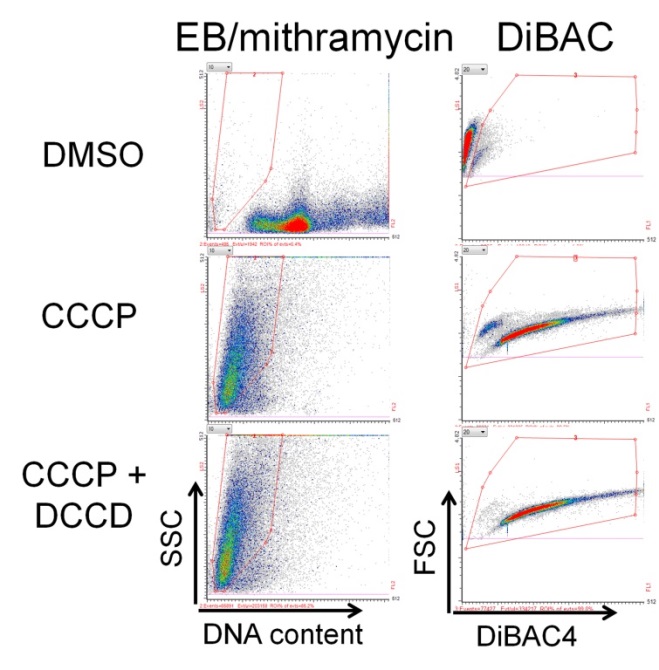


**Supplementary Fig. 11.** DCCD cannot inhibit CCCP-induced membrane depolarization and chromosome degradation. Exponential phase cultures were treated with DMSO, CCCP, CCCP+DCCD for 6 h, respectively. Then, the sample were stained with EB/mithramycinA and DiBAC4, respectively and analyzed by flow cytometry. The results were shown in FL2 (DNA content)-SSC and FL1 (DiBAC)-FSC cytograms.
